# *Topoisomerase II alpha *gene copy loss has adverse prognostic significance in *ERBB2*-amplified breast cancer: a retrospective study of paraffin-embedded tumor specimens and medical charts

**DOI:** 10.1186/1756-8722-1-12

**Published:** 2008-08-14

**Authors:** Lydia Usha, Bita Tabesh, Larry E Morrison, Ruta D Rao, Kris Jacobson, April Zhu, Sanjib Basu, John S Coon

**Affiliations:** 1 Division of Hematology and Oncology, Department of Medicine, Rush University; Chicago, Illinois, 60612, USA; 2 Abbott Molecular Inc. Des Plaines, Illinois, USA; 3 Midwest Palliative and Hospice Care Center, Glenview, Illinois, USA; 4 Division of Statistics, Northern Illinois University, De Kalb, Illinois, USA; 5 Department of Pathology, Rush University; Chicago, Illinois, 60612, USA

## Abstract

**Background:**

Amplification of the *ERBB2 *(*Her-2/neu*) oncogene, which occurs in approximately 25% of breast carcinomas, is a known negative prognostic factor. Available data indicate that a variable number of nearby genes on chromosome 17q may be co-amplified or deleted, forming a continuous amplicon of variable size. In approximately 25% of these patients, the amplicon extends to the gene for *topoisomerase II alpha *(*TOP2A*), a target for anthracyclines. We sought to understand the significance of these associated genomic changes for breast cancer prognosis and predicting response to therapy.

**Methods and patients:**

Archival tissue samples from 63 breast cancer patients with *ERBB2 *amplification, stages 0–IV, were previously analyzed with FISH probes for genes located near *ERBB2*. In the present study, the clinical outcome data were determined for all patients presenting at stages I–III for whom adequate clinical follow up was available.

**Results:**

Four amplicon patterns (Classes) were identified. These were significantly associated with the clinical outcome, specifically, recurrence of breast cancer. The Amplicon class IV with deleted *TOP2A *had 67% (6/9) cases with recurrence, whereas the other three classes combined had only 12% (3/25) cases (p-value = 0.004) at the time of last follow-up. *TOP2A *deletion was also significantly associated with time to recurrence (p-value = 0.0002). After adjusting for age in Cox regression analysis, the association between *TOP2A *deletion and time to recurrence remains strongly significant (p-value = 0.002) whereas the association with survival is marginally significant (p-value = 0.06).

**Conclusion:**

*TOP2A *deletion is associated with poor prognosis in *ERBB2*-amplified breast carcinomas. Clarification of the mechanism of this association will require additional study.

## Background

The *ERBB2 *(*Her-2/neu*) oncogene is amplified and over-expressed in 25% of invasive breast carcinomas [1-4]. In general, *ERBB2 *amplification confers an unfavorable prognosis, although its significance is less than that of the traditional prognostic factors – stage and grade. Also, it seems that the prognosis and response to therapy varies considerably within the spectrum of *ERBB2*-amplified breast carcinomas (BC), indicating that they are biologically heterogeneous. The first targeted anti-neoplastic agent, Trastuzumab (Herceptin©), a monoclonal antibody to ERBB2, produces a response in approximately 15% of heavily pretreated patients with metastatic BC as a single agent [5] and in combination with chemotherapy improved the overall survival of patients with metastatic BC by 5 months [6]. It has recently been shown to decrease the risk of BC recurrence by about 50% in patients with *ERBB2 *amplified tumors in the adjuvant setting [7]. Unfortunately, only a fraction of patients with *ERBB2*-amplified breast carcinomas respond to Trastuzumab, further evidence for heterogeneity among these tumors.

The *ERBB2 *oncogene is located at the 17q12 chromosomal locus. Many genes located close to *ERBB2 *on 17q12-q21 are known or suspected to play a role in carcinogenesis, and specifically, in breast carcinogenesis. Previous studies demonstrated that the negative effect on the prognosis of BC attributed to *ERBB2 *amplification could, in fact, be due to co-amplification of the region adjacent but telomeric to *ERBB2 *[8]. One of the genes located in this region is *Topoisomerase IIA *(*TOP2A*). It has been demonstrated that amplification of *ERBB2 *in BC cell lines and in primary breast tumors is associated with simultaneous amplification or deletion of the *TOP2A *gene [9-14]. Also, it has been shown that in BC, amplification of *ERBB2 *correlates with overexpression of *TOP2A *[15], but it cannot predict it [11]. Some authors argue that ERBB2 and TOP2A overexpression could be independent prognostic factors of poor survival in BC [16].

Topoisomerases are nuclear enzymes that modulate the topology of DNA by modifying the tertiary structure of the double helix. TOP2A is a 170-kD protein that binds to DNA, forming the cleavable complex, which allows intertwined replicated DNA strands to physically separate at the end of mitosis [17]. TOP2A is more highly expressed in rapidly proliferating cells, and expression is limited to the S to G2/M phases of cell cycle. TOP2A is a molecular target for some important anticancer drugs, including anthracyclines, which are the key chemotherapeutic agents in the treatment of BC. Anthracyclines stabilize the TOP2A cleavable complex and inhibit TOP2A catalytic activity (ibid). Therefore, it has been suggested that the empiric observation that *ERBB2*-amplified BCs respond relatively well to anthracycline-based chemotherapy [18-23] is due to co-amplification of *TOP2A *[24-28]. These data suggest that co-amplification of at least one of the genes adjacent to *ERBB2 *can play a role in the response to a specific chemotherapeutic agent widely used in the treatment of BC. The significance of amplification or deletion of other genes adjacent to *ERBB2 *remains to be determined. *TOP2A *aberrations (amplification or deletions) occur in less than 10% of *ERBB2 *non-amplified breast tumors [29]. This indicates that *ERBB2 *amplification could be the primary genetic event involving chromosome 17q in breast carcinogenesis and the *TOP2A *alterations are secondary events.

We showed previously that amplification of *ERBB2 *and nearby genes appears to form a single amplicon of variable size, without intervening normal or deleted segments [30], consistent with the break-fusion-bridge model for gene amplification [30,31], where recurrent double-stranded DNA breaks occur at vulnerable sites which become starting points for further amplifications or telomeric deletions. We systematically analyzed the amplification patterns of the region telomeric to *ERBB2*, using a series of fluorescence *in-situ *hybridization (FISH) probe sets. We determined that there are significant variations of the amplicon size, and that *TOP2A *can be amplified, normal or deleted. Here we conducted a retrospective study to determine whether the amplicon pattern, including amplification or deletion of *TOP2A *correlates with clinico-pathologic characteristics of breast tumors, markers of proliferation, and the clinical outcome of patients with *ERBB2*-amplified BC.

## Results

### Chromosome 17q gene copy abnormality patterns in *ERBB2*-amplified BCs

**Figure 1:**
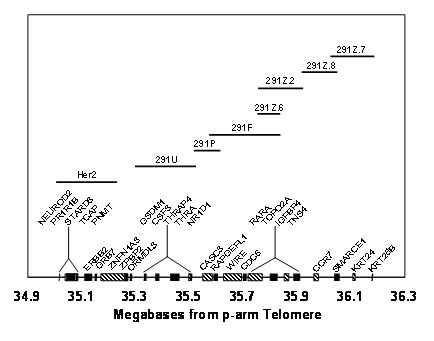
**Diagram showing location of FISH probes and selected genes on chromosome 17**. The locations of the 6 mapping probes and LSI^® ^Her2 probe pictured in Figure 1 are based on the probe sequences in the May 2006 assembly of the human genome browser on the University of California, Santa Clara web site .

We showed previously that BCs with *ERBB2 *amplification have variable amplification and deletion of genes telomeric to *ERBB2 *[30]. This was demonstrated with the Vysis *ERBB2 *probe and 7 other FISH probes covering an adjacent 889 kb telomeric region (Figure 1). The amplified region (amplicon) may extend to or even beyond the *TOP2A *gene, contained within the *291F*, *291Z.6*, *291Z.2 *probes, and appears to be continuous in 90% of cases studied, without intervening regions of normal copy number or deletion. If deleted segments are present, they begin just telomeric to the amplified segment, and almost always involve the *TOP2A *gene. In this study, the status of the *291Z.2 *probe was used to assess the copy number of the *TOP2A *gene because: 1) the gene is more centrally located within the *291Z.2 *sequence, as opposed to the 291F probe for which *TOP2A *is located near its telomeric end (furthest from *ERRB2*), and 2) *291Z.2 *targets a larger region than *291Z.6*, thereby providing a brighter signal that would be more accurately visualized in poorer quality specimens. The disadvantage of the *TOP2A *sequence lying near the terminus furthest from *ERBB2 *in the *291F *targeted region is that amplicons can terminate just centromeric to *TOP2A *but still contain the majority of the *291F *sequence, producing *291F *signals bright enough to be counted as amplified, and misrepresenting the *TOP2A *gene status.

Here, we have classified the17q gene copy abnormalities of 63 BC, including 9 cases with only DCIS, all with amplification of *ERBB2 *by FISH, into 4 categories of similar frequency, to permit comparison with other tumor attributes and clinical outcome:

**Class I**: *ERBB2 *and *TOP2A *both amplified (25.4% of total);

**Class II**: Only *ERBB2 *amplified (no other mapping probe loci amplified), *TOP2A *normal (23.8% of total),

**Class III**: *ERBB2 *and neighboring mapping loci amplified, *TOP2A *normal (25.4% of total),

**Class IV**: *ERBB2 *amplified but *TOP2A *deleted (25.4% of total).

### Associations between Amplicon Class, clinical and pathologic characteristics of breast tumors

Tests for association of Amplicon Class and accepted descriptors of breast carcinoma for all 63 patients are summarized in Table 1. No significant associations between Amplicon Class and presence of invasion, histological type, grade, stage, patient age, or hormone receptor status were found.

**Table 1:** Clinical and pathologic characteristics of breast tumors by Amplicon class

**Tumor Properties**		**Amplicon Class, number of patients**				
		**Class I**	**Class II**	**Class III**	**Class IV**	**p-value**

Invasion	present	14	12	14	14	0.913
	DCIS only	2	3	2	2	
Type (invasive)	Ductal	12	12	14	14	0.115
	Lobular	2	0	0	0	
Grade (invasive)	II	5	2	1	4	0.146
	III	9	10	13	10	
Clinical Stage	I	5	6	6	4	0.336
	II	4	5	5	5	
	III	2	2	1	5	
	IV	3	0	1	0	
Age	under 50	6	6	7	9	0.720
	over 50	10	9	9	7	
Hormone receptors	ER+/PR+	4	3	3	3	0.710
	ER+/PR-	4	2	5	3	
	ER-/PR+	2	1	0	0	
	ER-/Pr-	4	6	6	8	

### Associations between *TOP2A *gene copy number, protein expression, and cell proliferation

We studied nuclear TOP2A expression by immunohistochemistry (IHC) to determine whether it paralleled the gene copy (FISH) studies. A comparison of the frequency of expression by tumor cell nuclei versus Amplicon Class did not show a significant association (p = 0.50), data not shown. Similarly, comparing TOP2A expression frequency versus the *TOP2A *gene categorized as amplified, normal or deleted, did not show a significant association (p = 0.38), data not shown.

**Figure 2:**
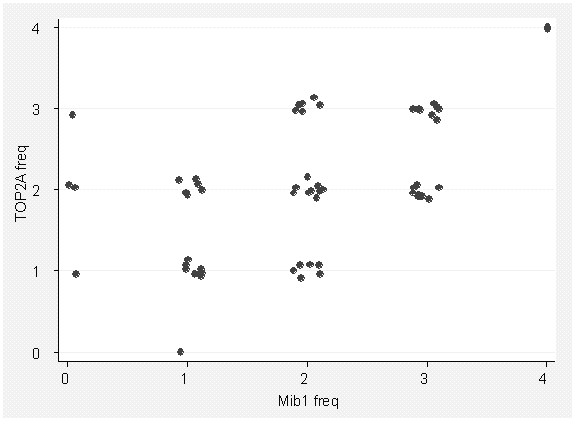
Association between frequency of expression of TOP2A and MIB1 in breast carcinoma cells.

TOP2A expression level has been reported to reflect the proliferation rate of tumors [17], and some authors have suggested that associations between TOP2A expression and/or gene amplification and the biological behavior of tumors occur on this basis (*ibid*). Here, we observed that TOP2A expression did show a modest association with expression of MIB1, a standard IHC assay for cell proliferation, correlation coefficient 0.54 (Figure 2).

The presence or absence of *TOP2A *amplification was not associated with the frequency of MIB1 expression (p = 0.79), nor was Amplicon Class associated with MIB1 frequency (p = 0.58).

### Association of BC clinical outcome with Amplicon Class and *TOP2A *gene copy number

The analysis of the clinical outcome data by chart review were obtained for 34 patients who presented with Stage I–III invasive BC and who had at least 18 months of clinical follow-up. The 9 patients with only DCIS were excluded from this analysis. Twenty-one other patients were also excluded, most often because follow-up was unobtainable, or they presented with Stage IV disease. Twenty patients received anthracycline-based therapy in the adjuvant setting and 3 in the neoadjuvant setting. The remainder never received anthracycline therapy, Table 2

**Table 2:** Clinical outcome is associated with Amplicon Class for patients with stage I–III invasive breast cancer

**Amplicon Class**	Clinical outcome and number of patients (Total N = 33)	
	
	NED or DNED	AWD or DOD
**Class I**	6 (4)	2 (0)
**Class II**	6 (5)	1 (0)
**Class III**	9 (9)	0
**Class IV**	3 (1)	6 (4)

**NED**: alive with no evidence of disease. **DNED**: died with no evidence of disease at least 24 months after diagnosis. **AWD**: Alive with breast cancer. **DOD**: died of breast cancer. NED and DNED were categorized as favorable outcome, and DOD and AWD as unfavorable for this analysis. Numbers in parentheses refer to the number of patients who received anthracycline-based therapy. The association between Amplicon class and clinical outcome is strongly significant, permutation based exact p-value = 0.007 from a chi-square test.

The Amplicon Class IV, with *TOP2A *deletion, had 67% (6/9) cases with unfavorable outcome, whereas the other three classes combined had only 12% (3/25) unfavorable outcomes. The association of *TOP2A *deletion with unfavorable outcome was strongly significant (p value = 0.004 from Fisher's exact test), whereas *TOP2A *amplification (Class I) did not confer a better outcome than normal *TOP2A *copy number (Class II and III). In contrast, no association was found between clinical outcome categories and expression of TOP2A (p = 0.66) or expression of MIB1 (p = 0.695). Clinical outcome categories were also not associated with tumor grade (p = 0.69), stage (p = 0.25), ER status (p = 0.78), PR status (p = 0.54, or patient age (p = 0.78). Five of the 9 unfavorable outcomes occurred in patients who had never received anthracycline therapy.

**Figure 3:**
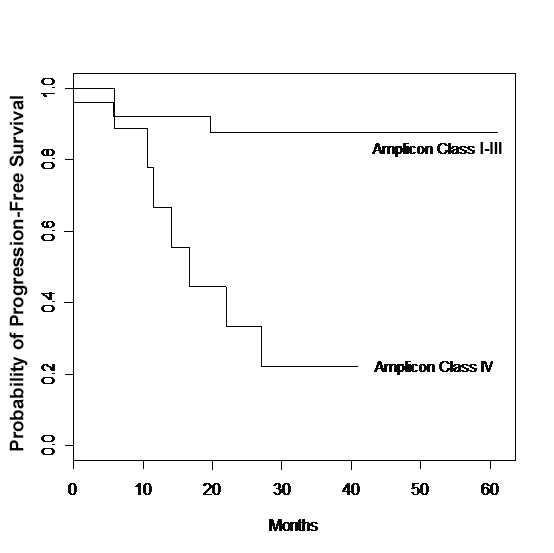
Time to recurrence for patients with stage I–III invasive breast cancer by Amplicon Class.

The Kaplan-Meier estimates of time to tumor recurrence for patients with *TOP2A *deletion versus others are shown (Figure 3). 56% of cases are estimated to recur by 18 months in the *TOP2A *deleted group, whereas only 8% are estimated to recur in the other patients. The difference in time to recurrence between these two groups is strongly statistically significant (p-value = 0.0002 from a log-rank test). Since the sample sizes are small, we also obtained the permutations based p-value of the log-rank test using the ExactRankTests package of the R statistical software, this latter p-value = 0.0006.

**Figure 4:**
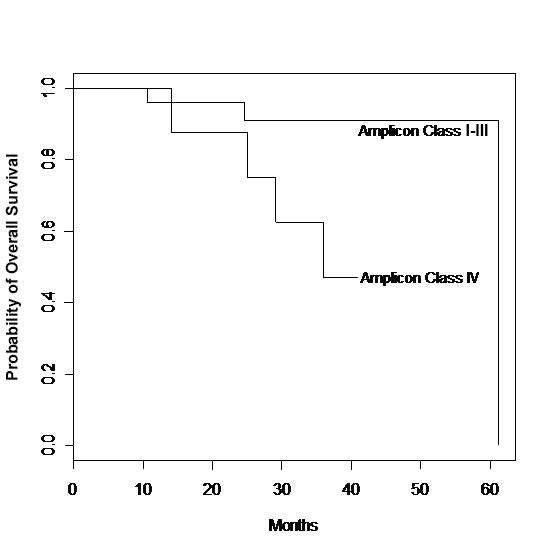
Survival for patients with stage I–III invasive breast cancer by Amplicon Class.

The Kaplan-Meier survival estimates of the *TOP2A *deletion group versus others are shown in Fig 4 (Figure 4). 44% (4/9) patients died during the follow-up in the *TOP2A *deletion group whereas 12% (3/25) died in the other group. The difference in survival between these two groups is statistically significant (p-value = 0.04 from a log-rank test and = 0.03 from the exact permutation based approach).

Due to the limited sample size, we considered multivariate analysis with *TOP2A *deletion and one additional covariate at a time. *TOP2A *deletion had significantly increased hazard of recurrence (HR = 8.6, p-value = 0.002) than the other group in a Cox proportional hazards regression analysis which also included age as a covariate. *TOP2A *deletion also had significantly increased hazard of recurrence after adjustment by grade (HR = 9.5, p-value = 0.002) and after adjustment by ER status (HR = 9.2, p-value = 0.002). The effect of *TOP2A *deletion on survival was marginally significant (HR = 5.2, p-value = 0.06) after adjustment by age. The effect of age, grade and ER were not at all significant in either one of these analyses.

Thus, we found that Amplicon Class with *TOP2A *deletion in *ERBB2*-amplified BC was associated with shorter time to tumor recurrence and significantly higher risk of cancer recurrence independent of other covariates.

## Discussion

This paper is the first attempt to relate the characteristics of the highly variable *ERBB2-TOP2A *amplicon in BC, categorized into clearly defined Amplicon Classes, to the phenotype of the tumors. The Amplicon Classes were not associated with the commonly used descriptors of breast neoplasia. Specifically there was no evidence that Amplicon Class was associated with the ability of tumor cells to invade normal tissue, since the Class distribution was similar in invasive carcinoma and DCIS. Amplicon Class also seemed not to vary with stage at presentation, or hormone receptor status. These observations suggest that Amplicon Class may be independent of the most useful classical prognostic markers.

*TOP2A *deletion emerged as a strong predictor of unfavorable outcome and shorter disease-free survival, whereas no significant association with *TOP2A *amplification was found. This is not inconsistent with our previous study of local tumor response in locally advanced BC, most of which did not have *ERBB2 *amplification, and all of which were treated with neoadjuvant anthracycline-based therapy [25]. *TOP2A *amplification was associated with tumor response in this study, but the patients and the end point were quite different than the present study. Comparing Amplicon Class II with Amplicon Class III, amplification of genes between *ERBB2 *and *TOP2A *was also not associated with outcome or time to recurrence in this study. We also assessed TOP2A expression by IHC, but we did not find a significant association with clinical outcome or time to recurrence. *TOP2A *deletion was not associated with significantly reduced expression of TOP2A. This raises the possibility that *TOP2A *deletion may be a marker for another genetic event, most likely involving a nearby gene, whose altered expression confers an adverse prognosis. In fact, the 291Z.2 probe contains all or part of at least 3 genes in addition to *TOP2A *(Figure 1). Identifying the significant gene will require studying more patients with *TOP2A *deletion at higher resolution. It is not possible to link the adverse prognostic significance of *TOP2A *deletion to anthracycline resistance in this group of patients, since 5 of the 9 patients with adverse outcomes did not receive anthracyclines. Given the existing evidence on the higher likelihood of *TOP2A *amplified tumors to respond to anthracyclines, this observation may indicate that *TOP2A *deletion is an unfavorable prognostic marker independent of chemotherapy, but the small number of patients does not allow drawing any definitive conclusion. The association between clinical outcome and anthracycline therapy (received vs. not received) was not significant (p = 0.4 from Fisher's exact test).

This study of Amplicon Class versus clinical outcome is based upon a small number of highly selected (*ERBB2*-amplified) BC patients with relatively short follow-up. These factors probably account for several unexpected findings in this study. Among all factors considered only *TOP2A *deletion was associated with survival. We have not observed the correlation between survival and stage, presumably because the follow-up was too short. The fact that all of the tumors had *ERBB2 *amplification may have obscured the association of other established prognostic factors, such as ER and PR with outcome. Nevertheless, the study is significant because it demonstrates that the recently recognized molecular heterogeneity of the *ERBB2 *amplification event may have clinical significance, although this finding requires confirmation in a larger group of patients. It has been assumed that the adverse effect of *ERBB2 *amplification is mediated largely or entirely via associated overexpression of the ERBB2 oncogene, but associated copy abnormalities in nearby genes may also be involved [32].

TOP2A has been studied in BC by other investigators, primarily because it is a marker of proliferation and a target for anthracyclines [33-35]. However, the information on the prognostic role of TOP2A is limited and the use of anthracyclines could be a confounding factor to assess it. Moreover, in previous studies [15,36] the prognostic role of TOP2A in BC was frequently studied by correlating its expression by IHC with the clinical outcome. More recent studies showed that TOP2A expression measured by is cell cycle-dependent and thus, indicates the number of proliferating cells rather than nuclear *TOP2A *status in a given tissue. Furthermore, cell proliferation potentially can be a source of bias in measuring TOP2A protein [37]. Since anthracyclines interact with TOP2A in the nucleus, it is important to determine TOP2A expression in the nucleus which can be done most accurately by determining *TOP2A *copy number as a surrogate marker. To illustrate this point Burgess *et al*. utilized RNA interference to knockdown *TOP2A *gene in the nucleus of lymphoma cells which resulted in increased resistance to an anthracycline, doxorubicin, but has not affected cell proliferation [28]. Our results confirmed that TOP2A expression in BC cells by IHC is associated with proliferation (MIB-1), but neither marker was associated with outcome in this group of patients. The association between *TOP2A *deletion and adverse outcome in these patients appears, therefore, to be unrelated to the cell proliferation rate.

A number of retrospective analyses of tissue specimens from earlier adjuvant clinical trials with anthracycline and non-anthracycline chemotherapy regimens have been recently published [9,19,27,38,39]. The body of literature supports the idea that *TOP2A *status predicts the response to anthracyclines in BC and it is possible that clinical benefit from anthracyclines is limited to patients with *ERBB2 *and *TOP2A *amplified tumors. However, the prognostic and predictive value of *TOP2A *deletion remains controversial. The occurrence of *TOP2A *deletion in BC has been well documented previously, primarily in tumors with *ERBB2 *amplification [31,37,40,41]. In the study of Hicks *et al*. [29] 50% of *ERBB2*-amplified breast tumors had *TOP2A *co-amplification and 16% had monoallelic deletion of *TOP2A*. In *ERBB2 *non-amplifed tumors, *TOP2A *was never amplified and in 5% of the tumors there were monoallelic deletions of both *ERBB2 *and *TOP2A *genes (*ibid*). In one published analysis of a large collection of primary breast tumor samples, *TOP2A *alterations were reported in 23% of all tumors, regardless of their *ERBB2 *status: 12% had *TOP2A *amplification and 11% had *TOP2A *deletion [38]. In this study both *TOP2A *amplification and deletion were associated with improved recurrence-free and overall survival if treated with anthracycline-based chemotherapy as opposed to a non-anthracycline regimen.

Our data confirm the results of the recently published retrospective analysis of tissue samples from the large adjuvant clinical trial [42] which have demonstrated that *TOP2A *aberrations, including both *TOP2A *amplifications and deletions, are significantly associated with shorter recurrence free and overall survival. A clear benefit from adjuvant anthracyclines was identified in women with *TOP2A *amplifications [38,29,42] and a non-significant trend for improved survival was observed in women with *TOP2A *deletions [42]. Thus, the *TOP2A *deletion in BC seems to confer a poor prognosis, but more studies are needed to elucidate the responsiveness of these tumors to anthracyclines.

Trastuzumab may be synergistic, additive or antagonistic in combinations with different chemotherapeutic agents. With the recent approval of Trastuzumab for the adjuvant treatment of BC and expanding its use, the importance of exploring molecular markers in the vicinity *of ERBB2 *is increased [43,44]. Slamon *et al*. [1] recently illustrated this point with the preliminary analysis of the BCIRG 006 clinical trial data [45]. These results suggest that patients with co-amplification of *TOP2A *comprise the subset of patients who benefit from anthracyclines in the adjuvant setting. Therefore, patients without *TOP2A *co-amplification may be better treated with combinations of non-anthracycline drugs with Trastuzumab, which would decrease the risk of cardiotoxicity. In fact, we recently reported that *ERBB2 *amplicons that did not extend to the *291Z.2 *(*TOP2A*) locus (Class II + Class III amplicons) were associated with improved response to trastuzumab relative to amplicons that included the *291Z.2 *locus (Class I amplicons) [46]. Although the combination of Trastuzumab and anthracyclines may seem to be very powerful against *ERBB2 *and *TOP2A *amplified BC, this combination is cardiotoxic. It would seem reasonable to search for new non-cardiotoxic inhibitors of TOP2A to combine with Trastuzumab. One of these agents, suberoylanilide hydroxamic acid (SAHA), is currently in clinical trials [47,48].

## Conclusion

The *TOP2A *deletion is associated with increased risk of BC recurrence and death from breast cancer in patient with *ERBB2 *amplified BC. Clarification of the mechanism of this association will require additional study.

## Materials and methods

### Hybridization Probes

The probes have been described in detail previously [30]. Single clones used for FISH probes were the following: *291U *(RP11 BAC 283i23), *291P *(RP5 PAC 1152A16), *291F *(CITB BAC 428H21), *291Z.2 *(RP11 BAC 58o9), *291Z.6 *(Genome Systems P1 # 611), *291Z.7 *(RP11 BAC 89A22), and *291Z.8 *(RP1 PAC 1028K7). The single clones lie within a contig beginning about 69 kb telomeric of the Vysis LSI^® ^*HER-2 *probe and extending for approximately 889 kb toward the 17q telomere (see Figure 1) Each *in situ *hybridization included 3 FISH probes directly labeled with different fluorophores: a peri-centromeric alpha satellite probe for chromosome 17 (Vysis^® ^SpectrumAqua™ CEP^® ^17; Abbott Molecular Inc, Des Plaines, IL), a probe for *ERBB2 *(Vysis SpectrumGreen™ LSI^® ^HER-2, Abbott Molecular) and one of 7 single-clone probes telomeric to *ERBB2 *labeled with SpectrumOrange™.

### Specimens

Fifty-four specimens from patients with invasive BC and 9 patients with ductal carcinoma *in situ*, without documented coexisting invasion, were obtained from the archives of the Pathology Department at Rush University Medical Center (Chicago, IL). They comprised left-over diagnostic material from patients seen between 1998 and 2003. There was sufficient archival material available for all of the patients included to ensure that the study did not exhaust the diagnostic tumor tissue. *ERBB2 *amplification was verified for all patients as part of this study, by use of the PathVysion^® ^FISH panel (Abbott Molecular). Paraffin blocks were sectioned at 5 μm thickness and mounted onto SuperFrost Plus^® ^positively charged slides (ThermoShandon, Pittsburgh, PA).

### Patients

Patients with *ERBB2*-amplified BC treated at Rush University Medical Center, Chicago, Illinois, between 1997 and 2004 were considered for the study. The study was approved by the Rush Institutional Review Board. Only patients for whom adequate archival pre-therapy tumor tissue and adequate clinical follow-up data were available were eligible for the study. The median follow up for the patients in this study was 31 months. The diagnosis of invasive BC in the archival material was confirmed by histological evaluation before further analysis. The clinical outcome data was obtained by chart review. No patients in this study received adjuvant Trastuzumab because they were treated before the approval of Trastuzumab for the adjuvant treatment of BC.

### *In Situ *Hybridization

The procedure has been described in detail previously [30]. Briefly, the specimens were prepared by immersion of the slides in Vysis Pretreatment Solution (Abbott Molecular) at 80°C for 10 minutes. The slides were then immersed in a solution of 4 mg pepsin (2500–3000 U/mg), rinsed in water, and dehydrated in 70%, 85%, and 100% ethanol. The slides were hybridized with the 3-color FISH probe solutions in a HYBrite™ automated co-denaturation oven (Abbott Molecular) and then immersed in 73°C 2 × SSC/0.3% NP40 for 2 minutes for removal of nonspecifically bound probe.

### Enumeration of FISH signals

Typically, 30–90 cells were enumerated in each specimen. The FISH slides were evaluated under a Zeiss Axioscope epi-fluorescence microscope (Carl Zeiss, Thornwood, NY). Only nuclei with morphology characteristic of malignant cells were counted. The mean number of signals per cell was calculated by totaling the number of signals from each cell and dividing by the number of cells counted. Mean *ERBB2 *and mapping probe signals per cell were divided by the mean *CEP17 *signals per cell to provide the ratio of *ERBB2*-to-chromosome 17 signals and mapping probe-to-chromosome 17 signals. A lower ratio cutoff of 0.75 and an upper ratio cutoff of 2.00 were selected for deletion and amplification, respectively [30].

### Immunohistochemistry

In preparation for antibody staining, paraffin sections (5 microns, freshly cut) were deparaffinized and rehydrated using standard technique. A microwave antigen retrieval method was then carried out in citrate buffer. The tissue was stained using a Ventana ES Histo-stainer (Ventana Medical Systems, Tucson, AZ), using supplied diaminobenzidine and avidin-biotin conjugate immunoperoxidase chemistry. Sections were stained for TOP2A with the JH2.7 monoclonal antibody from Lab Vision Corp. (Fremont, CA) at a dilution of 1:100 and MIB1 with the Ki-S5 antibody (Dakcytomation, Carpeneria, CA), dilution 1:50. A single block from the pre-therapy biopsy was selected for analysis for each patient on the basis of having the greatest area of well-preserved tumor. Immunostaining frequency of all tumor cells on each slide was scored subjectively on a scale of 0 to 4 (actual cell counting was not performed) without knowledge of clinical patient data, as previously described [49]. Less than 1% positive tumor cells were scored as 0, 1–10% as 1, 11–35% rated 2, 36 – 70% rated 3 and over 70% rated 4 on the scale. Tumor cell staining intensity was also scored on a scale of 0 to 4, but was found to be so closely related to frequency that it was not further considered in this study. Only nuclear staining was considered for TOP2A.

### Statistical analysis

Fisher's exact tests and Chi-square tests were used to measure the significance of the association between pairs of categorical variables such as those between the amplicon class, patient descriptors, molecular variables and the clinical outcome of evidence of disease. Permutation based exact p-value were used for these tests since they are more appropriate for small sample size. Specimens were divided into 4 categories based on the FISH data as explained below. Immunohistochemical expression was divided into three categories: overexpressed (frequency 3+ and 4+), expressed (frequency 1+ and 2+) and undetected (frequency 0).

Time to recurrence and overall survival were measured as months from the start of treatment to the time of tumor recurrence, death or last follow-up. Survival curves were estimated by the Kaplan-Meier method and are compared by the log-rank test. Due to the relatively small sample sizes, exact permutation based p-values, available in the R statistical software, are reported for the log-rank test. Age adjusted time to recurrence and time to survival are analyzed using the Cox proportional hazards regression. SAS version 9.0 and the R statistical software were used in the data analysis. All reported p-values are two-sided.

## List of Abbreviations

BC: Breast carcinoma; TOP2A: Topoisomerase II alpha; FISH:  Fluorescence *In-Situ *Hybridization; NED: No evidence of disease; AWD:  Alive with recurrent breast cancer; DOD: Died of breast cancer; DNED:  Dead with no evidence of disease at least 24 months after diagnosis; ER:  Estrogen receptor;  PR: Progesterone receptor; IHC:  Immunohistochemistry.

## Competing interests

Larry E. Morrison and Kris Jacobson are employees of Abbott Molecular Inc. John S. Coon has received research funding from the same company.

## Authors' contributions

LU helped to plan the study and wrote the manuscript. BT and RR gathered and interpreted clinical data. LM and KJ performed the FISH analysis and interpreted the data. AZ and SB performed statistical analysis and helped to format and interpret the data. JC helped to plan the study and contributed significantly to writing the manuscript.

## References

[B1] Slamon DJ, Godolphin W, Jones LA, Holt JA, Wong SG, Keith DE, Levin WJ, Stuart SG, Udove J, Ullrich A (1989). Studies of the HER-2/neu proto-oncogene in human breast and ovarian cancer. Science.

[B2] Revillion F, Bonneterre J, Peyrat JP (1998). ERBB2 oncogene in human breast cancer and its clinical significance. Eur J Cancer.

[B3] Dickson RB, Pestell RG, Lippman ME, DeVita VT, Hellman S, Rosenberg SA (2005). Cancer of the Breast. Cancer Principles & Practice of Oncology.

[B4] Berns EM, Foekens JA, Van Staveren IL, Van Putten WL, de Koning HY, Portengen H, Klijn JG (1995). Oncogene amplification and prognosis in breast cancer: relationship with systemic treatment. Gene.

[B5] Cobleigh MA, Vogel CL, Tripathy D, Robert NJ, Scholl S, Fehrenbacher L, Wolter JM, Paton V, Shak S, Lieberman G, Slamon DJ (1999). Multinational study of the efficacy and safety of humanized anti-HER2 monoclonal antibody in women who have HER2-overexpressing metastatic breast cancer that has progressed after chemotherapy for metastatic disease. J Clin Oncol.

[B6] Slamon DJ, Leyland-Jones B, Shak S, Fuchs H, Paton V, Bajamonde A, Fleming T, Eiermann W, Wolter J, Pegram M, Baselga J, Norton L (2001). Use of chemotherapy plus a monoclonal antibody against HER2 for metastatic breast cancer that overexpresses HER2. N Engl J Med.

[B7] Piccart-Gebhart MJ, Procter M, Leyland-Jones B, Goldhirsch A, Untch M, Smith I, Gianni L, Baselga J, Bell R, Jackisch C, Cameron D, Dowsett M, Barrios CH, Steger G, Huang CS, Andersson M, Inbar M, Lichinitser M, Lang I, Nitz U, Iwata H, Thomssen C, Lohrisch C, Suter TM, Ruschoff J, Suto T, Greatorex V, Ward C, Straehle C, McFadden E, Dolci MS, Gelber RD, Herceptin Adjuvant IHERA. Trial Study Team (2005). Trastuzumab after adjuvant chemotherapy in HER2-positive breast cancer. N Engl J Med.

[B8] Kauraniemi P, Kuukasjarvi T, Sauter G, Kallioniemi A (2003). Amplification of a 280-kilbase core region at the ERBB2 locus leads to activation of two hypothetical proteins in breast cancer. Am J Pathol.

[B9] Di Leo A, Gancberg D, Larismont D, Tanner M, Jarvinen T, Rouas G, Dolci S, Leroy JY, Paesmans M, Isola J, Piccart MJ (2002). HER-2 amplifications and topoisomerase IIα gene aberrations with predictive markers in node-positive breast cancer patients randomized treated with an anthracycline-based therapy or with cyclophosphamide, methotrexate and 5-fluoroouracil. Clin Cancer Res.

[B10] Di Leo A, Isola J (2003). Topoisomerase IIα as a marker predicting the efficacy of anthracyclines in breast cancer: are we at the end of the beginning. Clin Breast Cancer.

[B11] Durbecq V, Paesmans M, Cardoso F, Desmedt C, DiLeo A, Chan S, Friedrichs K, Pinter T, Van Belle S, Murray E, Bodrogi I, Walpole E, Lesperance B, Korec S, Crown J, Simmonds P, Perren TJ, Leroy JY, Rouas G, Sotiriou C, Piccart M, Larsimont D (2004). Topoisomerase-IIα expression as a predictive marker in a population of advanced breast cancer patients randomly treated either with single-agent doxorubicin or single-agent docetaxel. Mol Cancer Ther.

[B12] Murthy SK, Magliocco AM, Demetrick DJ (2005). Copy number analysis of c-erb-B2 (HER-2/neu) and topoisomerase IIalpha genes in breast carcinoma by quantitative real-time polymerase chain reaction using hybridization probes and fluorescence in situ hybridization. Arch Pathol Lab Med.

[B13] Olsen KE, Knudsen H, Rasmussen BB, Balslev E, Knoop A, Ejlertsen B, Knoop A, Ejlertsen B, Nielsen KV, Schonau A, Overgaard J, Danish Breast Cancer Co-operative Group (2004). Amplification of HER2 and TOP2A and deletion of TOP2A genes in breast cancer investigated by new FISH probes. Acta Oncol.

[B14] Jarvinen TA, Liu ET (2003). HER-2/neu and topoisomerase IIalpha in breast cancer. Breast Cancer Res Treat.

[B15] Depowski PL, Rosenthal SI, Brien TP, Stylos S, Johnson RL, Ross JS (2000). Topoisomerase IIα expression in breast cancer: correlation with outcome variables. Modern Pathology.

[B16] Fritz P, Cabrera CM, Dippon J, Gerteis A, Simon W, Aulitzky WE, Kuip H van der (2005). C-erbB2 and topoisomerase IIalpha protein expression independently predict poor survival in primary human breast cancer: a retrospective study. Breast Cancer Res.

[B17] Takimoto CH, DeVita VT, Hellman S, Rosenberg SA (2005). Topoisomerase interactive agents. Cancer Principles & Practice of Oncology.

[B18] Arpino G, Ciocca DR, Weiss H, Allred DC, Daguerre P, Vargas-Roig L, Leuzzi M, Gago F, Elledge R, Mohsin SK (2005). Predictive value of apoptosis, proliferation, HER-2, and topoisomerase IIalpha for anthracycline, chemotherapy in locally advanced breast cancer. Breast Cancer Res Treat.

[B19] Cardoso F, Durbecq V, Larsomont D, Paesmans M, Leroy YJ, Rouas G, Sotiriou C, Renard N, Richard V, Piccart MJ, Di Leo A (2004). Correlation between complete response to anthracycline-based chemotherapy and topoisomerase IIα gene amplification and protein overexpression in locally advanced/metastatic breast cancer. Int J Oncol.

[B20] Colozza M, Sidoni A, Mosconi AM, Cavaliere A, Bisagni G, Gori S, De Angelis V, Frassoldati A, Cherubini R, Bian AR, Rodino C, Mazzocchi B, Mihailova Z, Bucciarelli E, Tonato M (2005). Italian Oncology Group for Clinical Research. HER2 overexpression as a predictive marker in a randomized trial comparing adjuvant cyclophosphamide/methotrexate/5-fluorouracil with epirubicin in patients with stage I/II breast cancer: long-term results. Clin Breast Cancer.

[B21] Dressler LG, Berry DA, Broadwater G, Gowan D, Cox K, Griffin S, Miller A, Tse J, Novotny D, Persons DL, Barcos M, Henderson IC, Liu ET, Thor A, Budman D, Muss H, Norton L, Hayes DF (2005). Comparison of HER2 status by fluorescence in situ hybridization and immunohistochemistry to predict benefit from dose escalation of adjuvant doxorubicin-based therapy in node-positive breast cancer patients. J Clin Oncol.

[B22] Moliterni A, Menard S, Valagussa P, Biganzoli E, Boracchi P, Balsari A, Casalini P, Tomasic G, Marubini E, Pilotti S, Bonadonna G (2003). HER2 overexpression and doxorubicin in adjuvant chemotherapy for resectable breast cancer. J Clin Oncol.

[B23] Piccart M, Lohrisch C, DiLeo A, Larsimont D (2001). The predictive value of HER2 in breast cancer. Oncology.

[B24] Park K, Kim J, Lim S, Han S (2003). Topoisomerase IIα (topoII) and HER2 amplification in breast cancers and response to preoperative doxorubicin chemotherapy. Eur J Cancer.

[B25] Coon JS, Marcus E, Gupta-Burt S, Seelig S, Jacobson K, Chen S, Renta V, Fronda G, Preisler HD (2002). Amplification and overexpression of topoisomerase IIα predict response to anthracycline-based therapy in locally advanced breast cancer. Clinical Cancer Research.

[B26] Arriola E, Rodriguez-Pinilla SM, Lambros MB, Jones RL, James M, Savage K, Smith IE, Dowsett M, Reis-Filho JS (2007). Topoisomerase II alpha amplification may predict benefit from adjuvant anthracyclines in HER2 positive early breast cancer. Breast Cancer Res Treat.

[B27] Schindlbeck C, Janni W, Shabani N, Kornmeier A, Rack B, Rjosk D, Gerber B, Braun S, Sommer H, Friese K (2005). Isolated tumor cells in the bone marrow (ITC-BM) of breast cancer patients before and after anthracyclin based therapy: influenced by the HER2- and Topoisomerase IIalpha-status of the primary tumor?. J Cancer Res Clin Oncol.

[B28] Burgess DJ, Doles J, Zender L, Xue W, Ma B, McCombie WR, Hannon GJ, Lowe SW, Hemann MT (2008). Topoisomerase levels determine chemotherapy response in vitro and in vivo. Proc Natl Acad Sci USA.

[B29] Hicks DG, Yoder BJ, Pettay J, Swain E, Tarr S, Hartke M, Skacel M, Crowe JP, Budd GT, Tubbs RR (2005). The incidence of topoisomerase II-alpha genomic alterations in adenocarcinoma of the breast and their relationship to human epidermal growth factor receptor-2 gene amplification: a fluorescence in situ hybridization study. Hum Pathol.

[B30] Jacobson KK, Morrison LE, Henderson BT, Blondin BA, Wilber KA, Legtor MS, O'Hare A, Van Stedum SC, Proffitt JH, Seelig SA, Coon JS (2004). Gene copy mapping of the ERBB2/TOP2A region in breast cancer. Genes Chromosomes Cancer.

[B31] Jarvinen TA, Tanner M, Barlund M, Borg A, Isola J (1999). Characterization of topoisomerase IIα gene amplification and deletion in breast cancer. Genes, Chromosomes and Cancer.

[B32] Kauraniemi P, Barlund M, Monni O, Kallioniemi A (2001). New amplified and highly expressed genes discovered in the ERBB2 amplicon in breast cancer by cDNA microarrays. Cancer Research.

[B33] MacGrogan G, Rudolph P, Mascarel IDI, Mauriac L, Durand M, Avril A, Dilhuydy JM, Robert J, Mathoulin-Pelissier S, Picot V, Floquet A, Sierankowski G, Coindre JM (2003). DNA topoisomerase IIα expression and the response to primary chemotherapy in breast cancer. Br J Cancer.

[B34] Martin-Richard M, Munoz M, Albanell J, Colomo L, Bellet M, Rey MJ, Tabernero J, Alonso C, Cardesa A, Gascon P, Fernandez PL (2004). Serial topoisomerase II expression in primary breast cancer and response to neoadjuvant anthracycline-based chemotherapy. Oncology.

[B35] Petit T, Wilt M, Velten M, Millon R, Rodier JF, Borel C, Mors R, Haegele P, Eber M, Ghnassia JP (2004). Comparative value of tumor grade, hormonal receptors, Ki-67, HER-2 and topoisomerase IIα status as predictive markers breast cancer patients treated with neoadjuvant anthracycline-based chemotherapy. Eur J Cancer.

[B36] Rudolph P, MacGrogan G, Bonichon F, Frahm SO, de Mascarel I, Trojani M, Durand M, Avril A, Coindre JM, Parwaresch R (1999). Prognostic significance of Ki-67 and topoisomerase IIα expression in infiltrating ductal carcinoma of the breast. a multivariate analysis of 863 cases. Breast Cancer Research.

[B37] Jarvinen TA, Tanner M, Rantanen V, Barlund M, Borg A, Grenman S, Isola J (2000). Amplification and deletion of topoisomerase IIα associate with erb-2 amplification and affect sensitivity to topoisomerase II inhibitor doxorubicin in breast cancer. Am J Pathol.

[B38] Knoop AS, Knudsen H, Balslev E, Rasmussen BB, Overgaard J, Nielsen KV, Schonau A, Gunnarsdottir K, Olsen KE, Mouridsen H, Ejlertsen B, Danish Breast Cancer Cooperative Group (2005). Retrospective analysis of topoisomerase IIa amplifications and deletions as predictive markers in primary breast cancer patients randomly assigned to cyclophosphamide, methotrexate, and fluorouracil or cyclophosphamide, epirubicin, and fluorouracil: Danish Breast Cancer Cooperative Group. J Clin Oncol.

[B39] Tanner M, Isola J, Wiklund T, Erikstein B, Kellokumpu-Lehtinen P, Malmstrom P, Wilking N, Nilsson J, Bergh J (2006). Topoisomerase IIalpha gene amplification predicts favorable treatment response to tailored and dose-escalated anthracycline-based adjuvant chemotherapy in HER-2/neu-amplified breast cancer: Scandinavian Breast Group Trial 9401. J Clin Oncol.

[B40] Jarvinen TA, Liu ET (2003). Topoisomerase IIα gene (TOP2A) amplification and deletion in cancer – more common than anticipated. Cytopathology.

[B41] Park K, Han S, Gwak GH, Kim HJ, Kim J, Kim KM (2006). Topoisomerase II-alpha gene deletion is not frequent as its amplification in breast cancer. Breast Cancer Res Treat.

[B42] Nielsen KV, Ejlertsen B, Moller S, Jorgensen JT, Knoop A, Knudsen H, Mouridsen HT (2008). The value of TOP2A gene copy number variation as a biomarker in breast cancer: Update of DBCG trial 89D. Acta Oncol.

[B43] Carlson RW, Brown E, Burstein HJ, Gradishar WJ, Hudis Ca, Loprinzi C, Mamounas EP, Perez EA, Pritchard K, Ravdin P, Recht A, Somio G, Theriault RL, Winer EP, Wolff AC, National Comprehensive Cancer Network (2006). NCCN Task Force Report: adjuvant therapy for breast cancer. J Natl Compr Canc Netw.

[B44] Romond EH, Perez EA, Bryant J, Suman VJ, Geyer CE, Davidson NE, Tan-Chiu E, Martino S, Paik S, Kaufman PA, Swain SM, Pisansky TM, Fehrenbacher L, Kutteh LA, Vogel VG, Visscher DW, Yothers G, Jenkins RB, Brown AM, Dakhil SR, Mamounas EP, Lingle WL, Kelin PM, Ingle JN, Wolmark N (2005). Trastuzumab plus adjuvant chemotherapy for operable HER2-positive breast cancer. N Engl J Med.

[B45] Press MF, Bernstein L, Sauter G, Zhou JY, Eiermann W, Pienkowski T, Crown J, Robert N, Bee V, Taupin H, Villalobos I, Seelig S, Pegram M, Slamon DJ (2005). Topoisomerase II-alpha gene amplification as a predictor of responsiveness to anthracycline-containing chemotherapy in the Cancer International Research Group 006 clinical trial of trastuzumab (herceptin) in the adjuvant setting [abstract]. 28th Annual San Antonio Breast Cancer Symposium.

[B46] Morrison LE, Jewell SS, Usha L, Blondin BA, Rao RD, Tabesh B, Kemper M, Batus M, Coon JS (2007). Effects of ERBB2 amplicon size and genomic alterations of chromosomes 1, 3, and 10 on patient response to trastuzumab in metastatic breast cancer. Genes, Chromosomes and Cancer.

[B47] Kim MS, Blake M, Baek JH, Kohlhagen G, Pommier Y, Carrier F (2003). Inhibition of histone deacetylase increases cytotoxicity to anticancer drugs targeting DNA. Cancer Res.

[B48] Marchion DC, Bicaku E, Daud AL, Richon V, Sullivan DM, Munster PN (2004). Sequence-specific potentiation of topoisomerase II inhibitors by histone deacetylase inhibitor suberoylanilide hydroxamic acid. J Cell Biochem.

[B49] Venkatesan TK, Kuropkat C, Caldarelli DD, Panje WR, Hutchinson JC, Chen S, Coon JS (1999). Prognostic significance of p27 expression in carcinoma of the oral cavity and oropharynx. Laryngoscope.

